# Impaired Sprouting and Axonal Atrophy in Cerebellar Climbing Fibres following *In Vivo* Silencing of the Growth-Associated Protein GAP-43

**DOI:** 10.1371/journal.pone.0020791

**Published:** 2011-06-10

**Authors:** Giorgio Grasselli, Georgia Mandolesi, Piergiorgio Strata, Paolo Cesare

**Affiliations:** 1 Santa Lucia Foundation (IRCCS), Rome, Italy; 2 National Institute of Neuroscience-Italy, University of Turin, Turin, Italy; Hertie Institute for Clinical Brain Research and German Center for Neurodegenerative Diseases, Germany

## Abstract

The adult mammalian central nervous system has a limited ability to establish new connections and to recover from traumatic or degenerative events. The olivo-cerebellar network represents an excellent model to investigate neuroprotection and repair in the brain during adulthood, due to its high plasticity and ordered synaptic organization. To shed light on the molecular mechanisms involved in these events, we focused on the growth-associated protein GAP-43 (also known as B-50 or neuromodulin). During development, this protein plays a crucial role in growth and in branch formation of neurites, while in the adult it is only expressed in a few brain regions, including the inferior olive (IO) where climbing fibres (CFs) originate. Following axotomy GAP-43 is usually up-regulated in association with regeneration. Here we describe an *in vivo* lentiviral-mediated gene silencing approach, used for the first time in the olivo-cerebellar system, to efficiently and specifically downregulate GAP-43 in rodents CFs. We show that lack of GAP-43 causes an atrophy of the CF in non-traumatic conditions, consisting in a decrease of its length, branching and number of synaptic boutons. We also investigated CF regenerative ability by inducing a subtotal lesion of the IO. Noteworthy, surviving CFs lacking GAP-43 were largely unable to sprout on surrounding Purkinje cells. Collectively, our results demonstrate that GAP-43 is essential both to maintain CFs structure in non-traumatic condition and to promote sprouting after partial lesion of the IO.

## Introduction

Reorganization of terminal arbors and synaptic remodelling, thought to underlie some aspects of learning and memory, occurs throughout life in the intact brain [1], [2] and plays a crucial role in recovery after brain injury [3]. An important mediator of structural plasticity of axonal fibres is the growth-associated protein GAP-43 [4]–[6]. The expression of GAP-43 is high in the brain during development and it declines in most neurons when mature synapses are formed. However in some brain regions a high expression of GAP-43 is maintained throughout life [7], [8] and it is suggested to play an important role in synaptic plasticity and synaptic vesicle release during adulthood [4]–[6], [9]. This is confirmed by studies performed on mice lacking one or both copies of *Gap-43* gene or expressing a point-mutated form, revealing alterations in well established learning and memory paradigms [10]–[14].

Previously it has also been shown that GAP-43 plays a major role in axonal sprouting. For instance, transgenic mice overexpressing GAP-43 in motoneurons exhibit both a spontaneous sprouting and an increased sprouting following block of neuromuscular transmission by botulinum toxin [15]. On the other hand, Purkinje cells (PCs) never express GAP-43 and show no sprouting after axotomy. However, in transgenic mice expressing GAP-43 selectively in PCs, sprouting appears both at the lesion site and along the intact axon surface, showing that the over-expression of GAP-43 is sufficient to induce sprouting [16]–[18]. We further investigated this point by assessing whether axonal sprouting is prevented by down-regulation of GAP-43 in neurons which constitutively express it and which are able to sprout.

Another aspect still poorly understood is whether GAP-43 plays any function in the structure of neurons of the adult brain under non-traumatic conditions, as observed during development.

Homozygotic knockout mice lacking *Gap-43* gene die early in the postnatal period [19], while brain development in heterozygotic knockout mice is profoundly affected, showing severe impairments of axonal pathfinding and in the formation of telencephalic commissures [11], [15], [19]–[21]. Hence, the possibilities for these transgenic animals to represent a proper model for studying GAP-43 during adulthood are very limited.

Here we describe the use of an *in vivo* gene silencing approach based on the injection of lentiviral particles encoding both a green fluorescent protein (GFP) and a specific short-hairpin RNA (shRNA) targeting *Gap-43* mRNA sequence. Using this technique we have investigated the structural role played by GAP-43 in axonal fibres of the adult brain under physiological conditions and its requirement in axonal sprouting. To this aim the cerebellar cortex provides an excellent model due to its high degree of structural plasticity [22]–[30]. The IO is among the regions retaining a high expression of GAP-43 throughout life [7], [8], [31]. Additionally, CFs, which are the terminal arbors of the axon of olivary neurons, innervate the dendrites of the PCs in a one-to-one relationship, displaying a well characterised three-dimensional organization [32], [33]. Previous studies investigating regeneration in the central nervous system have also shown that, following a subtotal lesion of the IO, the surviving CFs display a remarkable collateral sprouting, leading to reinnervation of the nearby located CF-deprived PCs [28], [34].

Here we show that specific silencing of GAP-43 in the IO of adult rodents leads to an atrophy of CF arborisation, to modifications of its synaptic varicosities and to a dramatic reduction of collateral sprouting after IO injury. In conclusion, these data demonstrate that in the cerebellum GAP-43 plays an important role in maintaining the complex structure of the CFs under physiological conditions. In addition, by showing *in vivo* that it is required for CF sprouting, they give a significant contribution to consolidate the idea that GAP-43 is an important target for promoting regeneration of the nervous system following injury.

## Results

### 1. *In-vivo* silencing of GAP-43 by lentiviral delivery of shRNA

**Figure 1:**
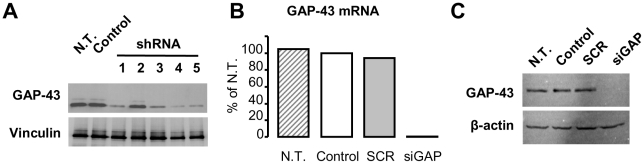
Silencing of GAP-43 in PC12 cells. (A) Western blot of GAP-43 (upper line) in NGF-differentiated PC12 cells not transduced (N.T.), transduced with GFP-only (control) or with one of the five silencing constructs (shRNA1-5). Vinculin is used as a loading control (lower line). (B) Real-time PCR showing *Gap-43* mRNA levels in PC12 cells not transduced (N.T.) or transduced with a highly concentrated (by double centrifugation) lentiviral preparation expressing GFP only (Control), shRNA4 (siGAP) or a scramble sequence (SCR). Two independent cultures per case; reference gene: ATP5B. (C) Western blot of GAP-43 in NGF-differentiated PC12 cells transduced with highly concentrated lentivirus. β-actin is used as a loading control (lower line).

In order to downregulate GAP-43 protein expression in olivary neurons we designed five candidate shRNA sequences (shRNA1-5) and cloned them into a GFP-encoding lentiviral vector. Silencing efficacy was evaluated by western-blot in rat pheochromocytoma PC12 cell line [35], [36] (Fig. 1A). These cells were chosen as they express high levels of GAP-43 following differentiation in dopaminergic neuron-like cells by NGF treatment [4].

High expression of shRNA, as achieved by the use of lentiviral vectors, can induce off-target effects leading to structural and functional modifications [37]. This is possibly due to the activation of an interferon response [38]. Therefore, in a series of control experiments, we included a scrambled sequence designed by randomizing the selected shRNA sequence in order to avoid any possible RNA target.

As shown in Fig. 1A, in cells treated with shRNA1, 3, 4 and 5, GAP-43 protein expression was significantly reduced, if compared to non-treated cells and control conditions. In particular, in cells treated with shRNA4, very low levels of expression were observed. These data were further confirmed at mRNA level by real-time PCR. Here a scrambled sequence (SCR) was tested against shRNA4 showing the specificity of the silencing effect (Fig. 1B). Increasing the titre of the viral stock, as obtained by a double-centrifugation protocol, allowed *in vitro* silencing of GAP-43 down to the detection limit when compared to non-treated cells or to cells treated with control or scrambled sequences (Fig. 1C). On the basis of these results all following experiments were performed using shRNA4 (renamed siGAP).

Viral preparations (siGAP, SCR and control) were stereotaxically injected into the IO of rats at post-natal day 19–22 (Supplementary Fig. S1), when cerebellar cortex has completed its development [39]. Rats were chosen for the possibility to induce a subtotal lesion of the IO by intraperitoneal injection of 3-acetylpyridine (3-AP), as this treatment was shown to elicit plastic changes in surviving CFs [28], [34].

**Figure 2:**
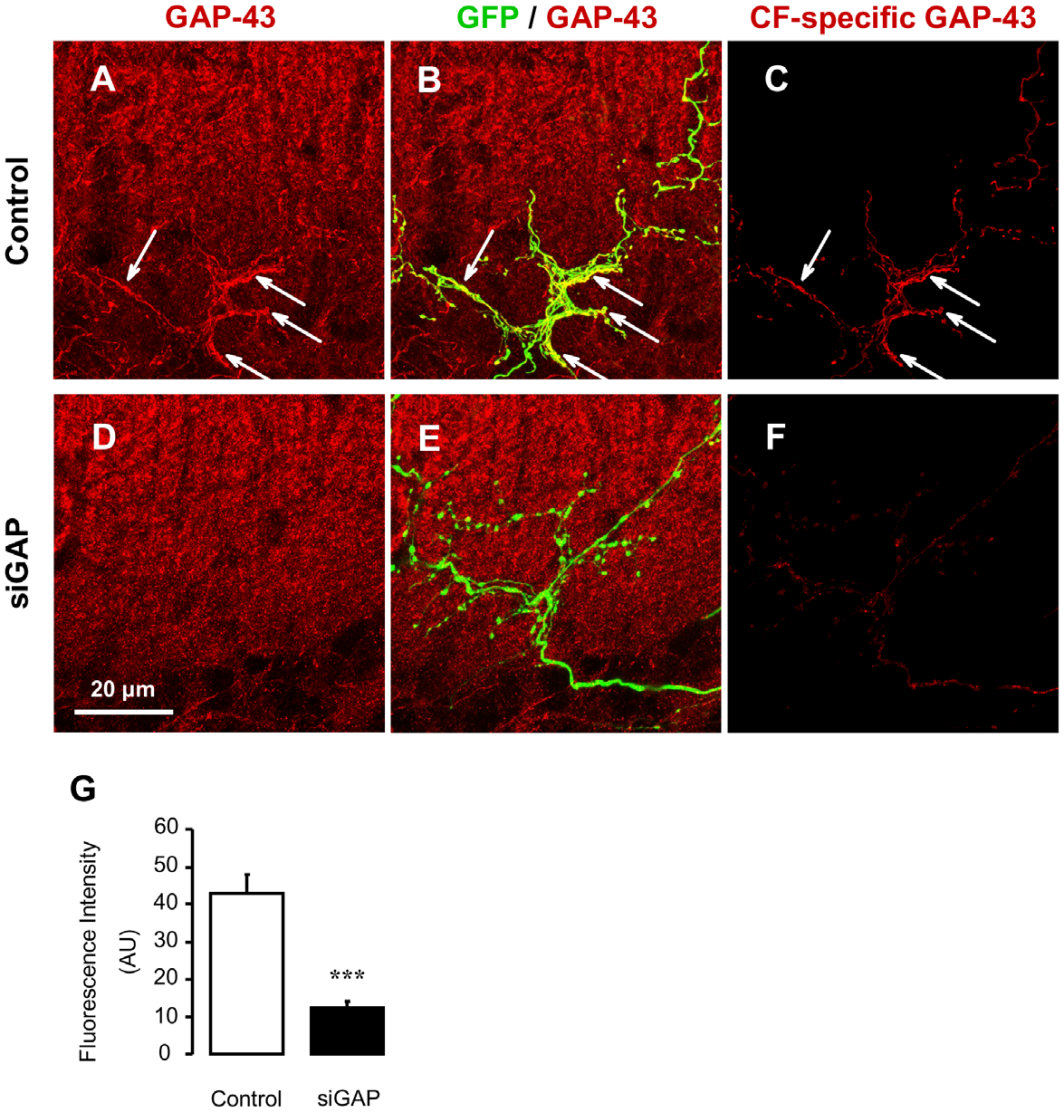
Silencing of GAP-43 in climbing fibres. (A–F) Projection of a series of optical sections from rats transduced with control (A–C) or siGAP viral particles (D–F). Images were obtained by confocal microscopy on cerebellar slices immunostained with anti-GAP-43 antibody (red). Only transduced CFs are labelled by GFP expression (green). (C, F) GAP-43 signal co-localizing with GFP has been isolated for each optical section and z-projected. High expression of GAP-43 is identified in control CFs (A–C, arrows), but not in siGAP CFs (D–F), demonstrating efficient *in vivo* silencing by siGAP sequence. (G) Quantification of GAP-43 immunofluorescence intensity in specifically identified CFs (N = 11 and 9; 3 animals per group; mean ± SEM; *** p<0.001).

The efficacy of GAP-43 silencing in olivary neurons was then verified *in vivo* by immunofluorescence on fixed cerebellar slices 3 weeks after the injection. GFP-expression in CFs was used as an unequivocal marker of transduction with the lentiviral vector. Laser scanning confocal microscopy was used to measure GAP-43 protein expression in CFs by specific colocalization with GFP fluorescence. Injection of siGAP lentiviral particles, compared to control, induced a dramatic reduction of GAP-43 expression, confirming effective *in vivo* silencing (8-bit brightness intensity arbitrary units: control = 41.0±4.1, siGAP = 14.5±2.0; p<0.001, N = 11 and 9 fibres respectively; Fig. 2).

### 2. Atrophy of climbing fibres

**Figure 3:**
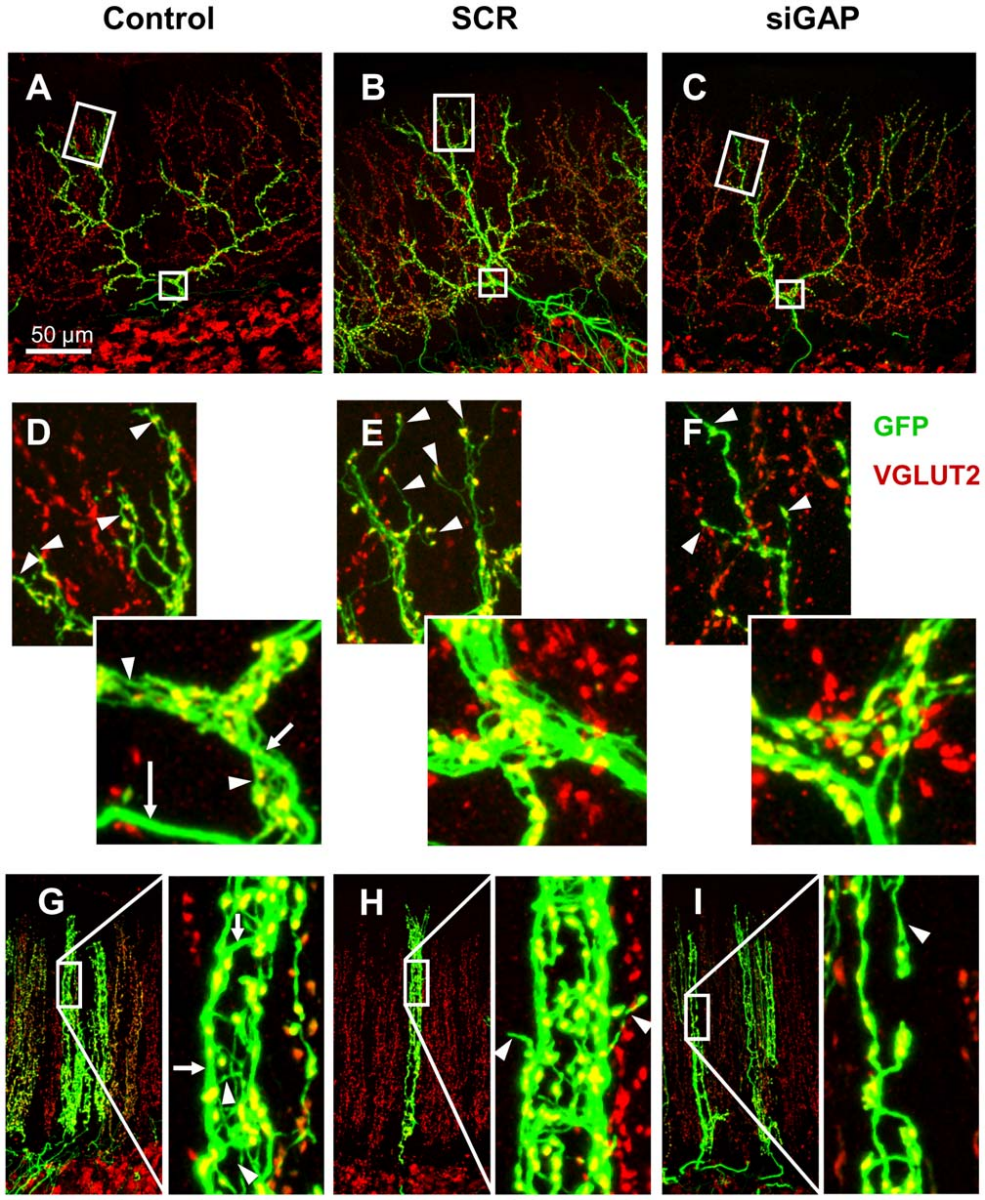
Morphology of rat CFs after silencing of GAP-43. Representative confocal images of rat parasagittal (A–F) and coronal (G–I) cerebellar sections, immunolabelled for VGLUT2 (in red). CFs were transduced with control (A, G), SCR (B, H) and siGAP sequences (C–I). (D–F) Details of the terminal and proximal tracts of CFs shown in A–C. The thick axonal stalk is pointed by arrows in D and G. Some of the numerous thin collaterals called tendrils are pointed by arrowheads in D–I. siGAP CFs are less branched in comparison to control and SCR CFs (A–C). At high magnification both on parasagittal and coronal view a reduction in the number of tendrils is evident in siGAP fibres (D–I), especially in terminal tracts (D–F). As a consequence, VGLUT2-positive varicosities appear reduced along the whole fibre (D–I).

The CF is composed by a thick axonal stalk from which many thin collaterals originate (namely tendrils; Fig. 3A, D, G). Some of these collaterals run parallel to the stalk on the PC dendrite while shorter ones form a net-like structure around the dendrite that appears particularly complex on the thickest and most proximal portion of the dendrite. In its most distal portions the CF is composed by fewer tendrils and the stalk decreases in diameter (Fig. 3A, D). These morphological features, clearly recognizable due to expression of GFP, are retained by CFs expressing SCR sequence, while fibres expressing siGAP sequence exhibit a characteristically different structure with fewer tendrils along their proximal and distal portions (Fig. 3A–F). A similar pattern can be observed on coronal sections (Fig. 3G–I) where a reduction of density of varicosities is particularly evident.

**Figure 4:**
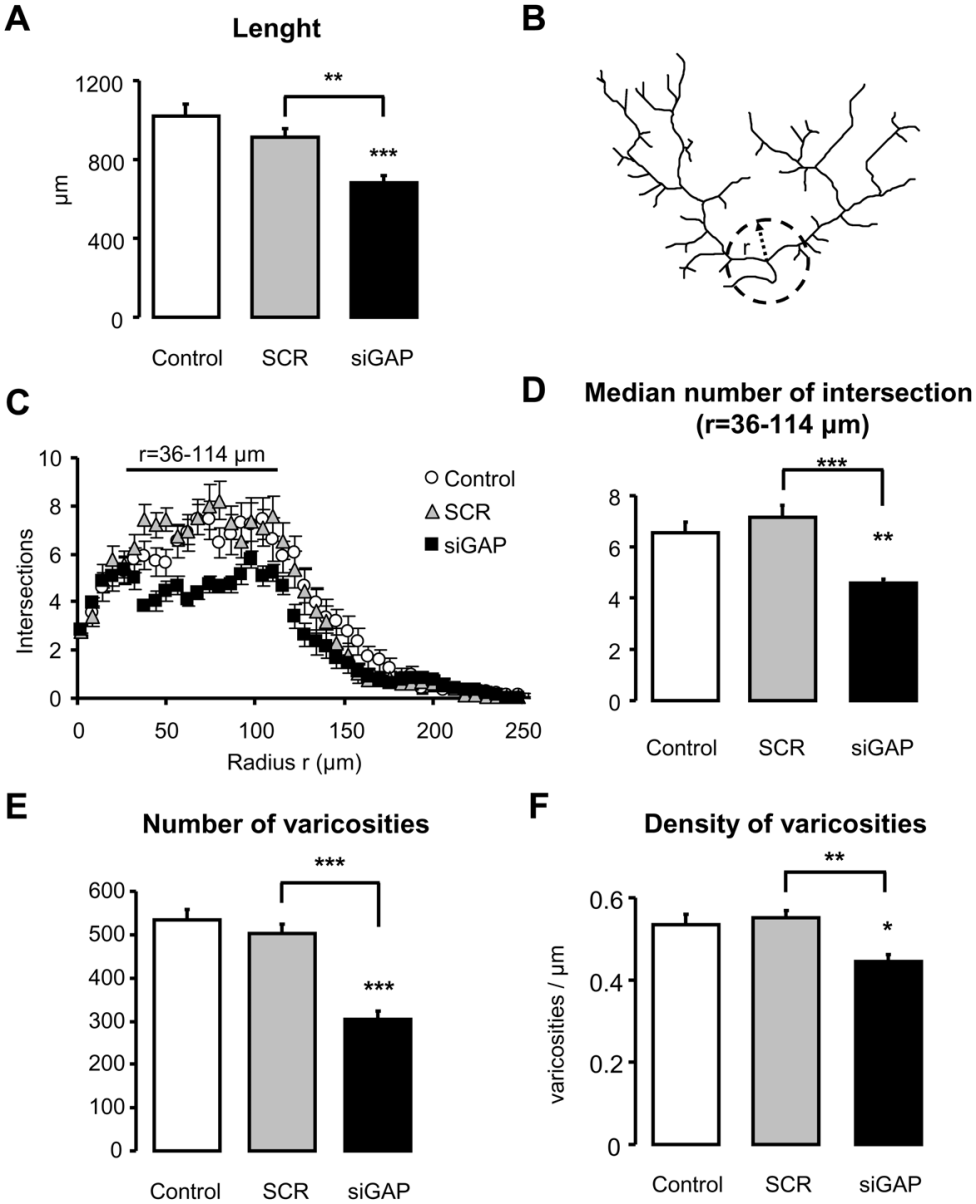
Morphometric analysis in juvenile rats reveals CF atrophy after GAP-43 silencing. (A) A significant reduction of CFs length is observed in siGAP compared to control and SCR conditions. (B) Schematic representation of Sholl's analysis for CF branches traced in Fig. 3A: complexity of branching was quantified as the number of intersections between the CF and a series of concentric circle. (C) Sholl's analysis shows the number of CF branches as a function of the distance from the main branching point in control, SCR and siGAP fibres (values every 6 µm are shown). (D) Number of branches as assessed by Sholl's analysis. For each fibre median value is obtained in the range from 36 to 114 µm, where its maximum is observed. (E) Number of varicosities and (F) density of varicosities per length unit (N = 14, 14 and 13; 3 animals per group; mean ± SEM; Tukey's post-hoc test: *p<0.05; ** p<0.01; *** p<0.001).

In order to quantify the changes of the CF arbor following GAP-43 down-regulation we first measured its total length. In control conditions the mean value was 1024±56 µm. Following transduction with SCR sequence we obtained a mean value of 911±43 µm, which was not statistically different from control. Conversely, a significant decrease was found in the length of GAP-43 deprived arbors, which had a mean value of 683±33 µm (p<0.001; post-hoc comparisons: siGAP vs. control, p<0.001; siGAP vs. SCR, p<0.01; N = 14, 14 and 13 CFs respectively control, SCR and siGAP CFs; Fig. 4A).

To obtain a bi-dimensional index of the structural complexity of the CFs in different conditions we measured branching fibres by Sholl's analysis (Fig. 4B) [40], [41]. A statistically significant decrease in the median number of intersections between 36 and 114 µm from the centre was present in the GAP-43 deprived CFs, relative to control and SCR conditions (control: 6.5±0.4; SCR = 7.2±0.5; siGAP = 4.6±0.2; p<0.001; post-hoc comparisons: siGAP vs. control p<0.01, siGAP vs. SCR, p<0.001; N = 14, 14 and 13 CFs respectively control, SCR and siGAP CFs; see Fig. 4C–D).

A further analysis was aimed at determining the number of varicosities in each CF arbor, identified either by their morphology or by VGLUT2 immunostaining [42]. As shown in Fig. 4E, their total number decreased significantly from a mean value of 544±23 in control fibres and 501±23 in SCR fibres to a value of 304±20 in siGAP fibres (p<0.001; post-hoc comparisons: siGAP vs. control and siGAP vs. SCR, p<0.001; N = 14, 14 and 13 CFs respectively control, SCR and siGAP CFs; Fig. 4E).

To assess whether the decrease in the number of varicosities was only due to the observed reduction of CF length or not, we measured the density of varicosities per µm of fibre length. As shown in Fig. 4F, while the density mean value was not statistically different between control and SCR, in GAP-43 deprived fibres a significant decrease was measured compared to both control and SCR conditions (control: 0.53±0.03; SCR = 0.55±0.02; siGAP = 0.44±0.02; p<0.01; post-hoc comparisons: siGAP vs. control p<0.05, siGAP vs. SCR, p<0.01; N = 14, 14 and 13 CFs respectively control, SCR and siGAP CFs; Fig. 4E).

**Figure 5:**
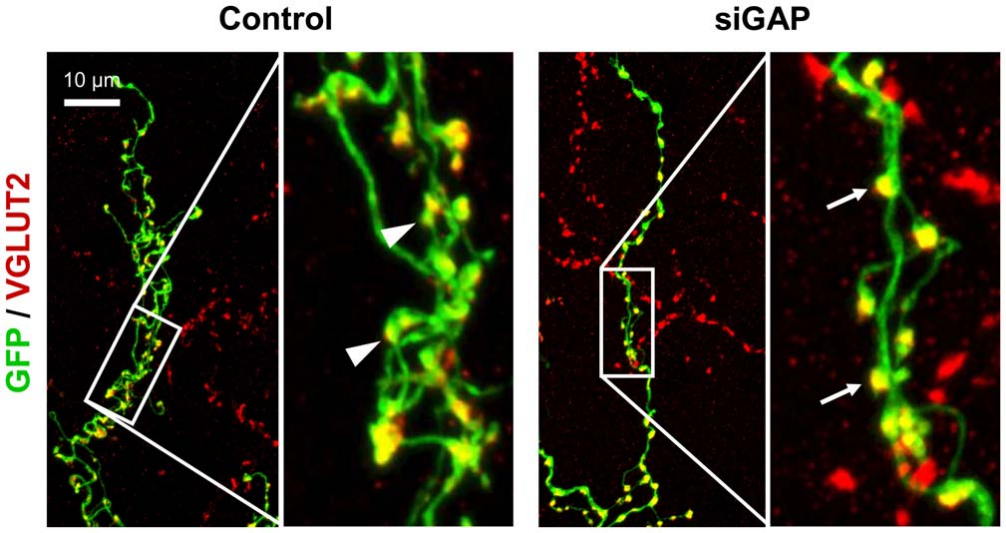
Morphology of varicosities in control and siGAP CFs in rats. Representative images of control and siGAP CFs acquired by confocal microscopy at high magnification (objective 63×, zoom 3×), showing larger VGLUT2-positive varicosities in siGAP fibres (arrows) compared to control. Small varicosities (arrowheads) are less frequent in siGAP fibres than in control. A reduction in the number of varicosities and tendrils is also detectable.

Varicosities are essential components of axons, as they contain most of the cytosolic and membrane-bound proteins involved in synaptic transmission. We observed that, following silencing of GAP-43, atrophic CFs have fewer small varicosities, while the remaining are larger and more regularly-shaped (Fig. 5).

During the period of GAP-43 silencing (between the third and sixth week of postnatal life) rat body growths, suggesting that depletion of GAP-43 may affect axonal growth during this time window. In order to investigate if the atrophic modification observed in CF was at least partially independent of axonal growth and age-related body growth we replicated the key experiments in older animals. For the different proportions of head bones in older rats, a proper exposure of the dorsal side of brain stem was technically not possible in a normal stereotaxical apparatus in these animals. However, this method of injection was easily adapted to adult mice (2–3 months old FVB mice) as it had the added bonus of opening up the possibility to extend our findings on the role played by GAP-43 in maintaining CF structure to other *in vivo* studies (such as in combination with genetically modified mouse models or with time-lapse imaging by multi-photon microscopy).

**Figure 6:**
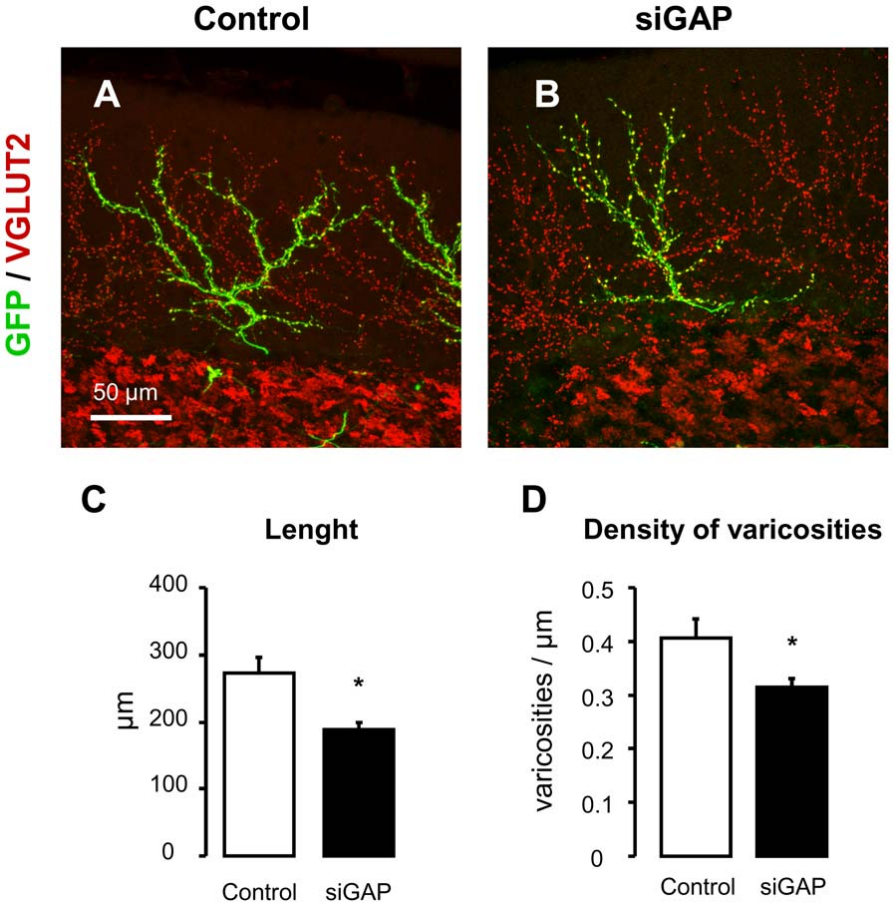
Morphometric analysis in adult mice confirms CF atrophy after GAP-43 silencing. Representative confocal images of mouse parasagittal cerebellar sections, immunolabelled for VGLUT2 (in red; A–B). CFs were transduced with control (A) and siGAP sequences (B). (C) Total length and (D) density of varicosities per length unit are reduced in siGAP CFs compared to control (N = 19 and 15, from 3 animals per group, mean ± SEM).

Using the same methodological approach used in rats, we observed also in mice a significant reduction in the length of CFs and the density of varicosities (length: control = 691±32 µm, siGAP = 598±27 µm, p<0.05; varicosities/µm: control = 0.41±0.04; siGAP = 0.32±0.01, p<0.05; N = 19 and 15; Fig. 6). This shows that GAP-43-dependent maintenance of CF structure is, at least partially, independent of age, suggesting that silencing of GAP-43 is able to induce a regressive modification of CF independent of axonal growth.

### 3. GAP-43 is crucial for CF sprouting induced by a subtotal lesion of the inferior olive

**Figure 7:**
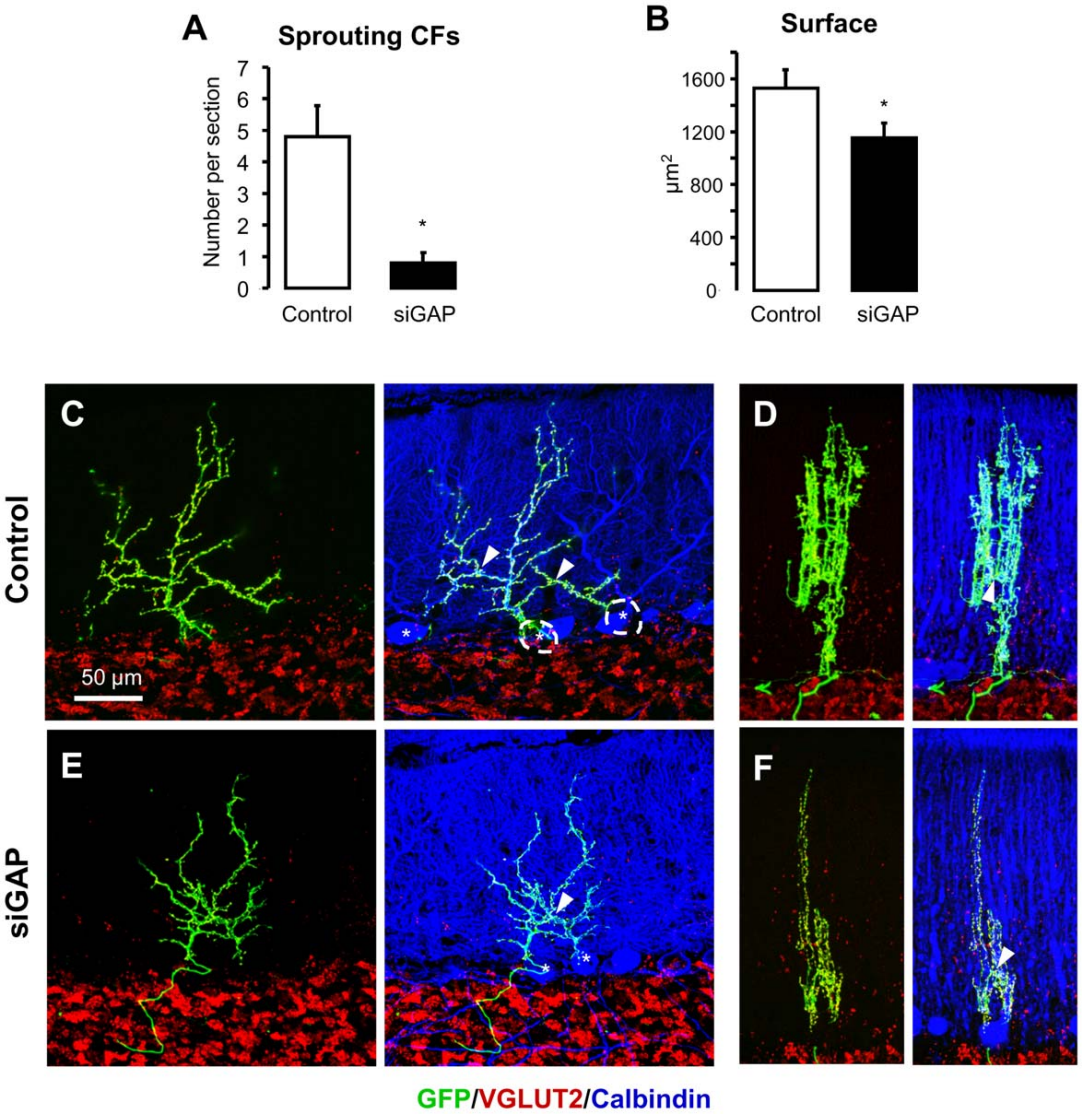
Collateral sprouting induced by sub-total lesion of the IO is inhibited by GAP-43 silencing. (A) Sprouting in GFP-positive CFs, induced by the sub-total lesion of the IO, is significantly reduced in rats treated with siGAP viral particles compared to controls (N = 3 and 5, respectively; number of slice analyzed per animal was 10, 17, 27 and 6, 6, 12, 6, 8; *p<0.05; mean ± SEM). (B) The total extension of those CFs still able to grow sprouts following GAP-43 silencing was also reduced as assessed by GFP labelling on coronal sections (N = 19 and 17 CFs, respectively, from 5 control and 3 siGAP animals; mean ± SEM; *p<0.05). (C, E) Sample images of sprouting CFs on sagittal and (E–F) coronal planes from control and siGAP rats 3 weeks after lesion of the IO (GFP, green; VGLUT2, red; Calbindin, blue). The almost complete lack of VGLUT2 labelled varicosities in molecular layer shows the degeneration of CFs due to 3-AP treatment. Asterisks and dotted lines indicate cell bodies of innervated PCs. Arrowheads indicate sprouting points for reinnervation of a nearby dendritic arbor. The absence of VGLUT2 labelling in the molecular layer indicates the degeneration of CFs originally surrounding the surviving CFs.

Collateral sprouting of CFs can be obtained in the rat following a subtotal lesion of the IO by means of an intraperitoneal injection of 3-AP. Surviving IO neurons exhibit collateral sprouting of the CF arbor in the molecular layer, where they can innervate one or more of the nearby located PCs deprived of their original CF afferent [28], [34]. In order to assess the *in vivo* requirement of GAP-43 in this process, we performed a subtotal lesion of the IO three weeks after injecting lentiviral particles encoding GFP (control) or both GFP and siGAP sequences. Following an additional 3 weeks, we confirmed neurotoxic effects of 3-AP on IO neurons by observing the presence of large areas, in the molecular layer, that were totally or sub-totally deprived of CF varicosities (identified by VGLUT2 staining; Fig. 7C–F). In contrast, animals that were not treated by 3-AP (Fig. 3) are characterized by an evenly dense distribution of VGLUT2-labelled varicosities throughout the whole molecular layer. Morphological analysis of the surviving CFs, both on sagittal and coronal planes revealed a picture that is consistent with previous reports [28]. Here, sprouting could be observed in one or more points in control CFs, both in sagittal and coronal sections (Supplementary Fig. S2). These points were identified as those where the CF was moving away from the dendrite of its own PC to contact the dendrite of a PC located either within the same sagittal plane or in an adjacent one (Supplementary Fig. S2A–B, inserts).

A difference in sprouting between control and GAP-43 silenced rats was then observed (Fig. 7). The total number of CFs showing collateral sprouting, as assessed on coronal sections, was significantly reduced in siGAP samples to a mere 16% of control (from 4.8±1 CFs per section in control animals to 0.8±0.6 in siGAP animals; p<0.05, N = 5 and 3 animals; the number of slices analyzed per animal was respectively 6, 6, 12, 6, 8 and 10, 17, 27), demonstrating *in vivo*, for the first time to our knowledge, that GAP-43 plays a crucial role in axonal sprouting in the cerebellum (Fig. 7A).

An additional issue was to quantify and compare the area occupied by those CFs undergoing sprouting in control rats and after GAP-43 silencing. The surface was measured on coronal sections based on GFP signal, and a significant reduction was observed in rats treated with siGAP viral particles compared to control (from 1530±137 µm^2^ to 1152±116 µm^2^, p<0.05, N = 19 and 17 CFs, from 5 control and 3 siGAP animals; Fig. 7B–F). This confirms that, at least in the cerebellum, GAP-43 plays an important role in establishing both the regenerative potential and the extension of axonal sprouting.

## Discussion

Several studies have shown, using transgenic mice, that overexpression of GAP-43 induces axonal sprouting [4], [43], suggesting that it represents an appealing target to promote central nervous system repair following brain injuries [3]. However, the role played by GAP-43 in adult uninjured brain still remains largely speculative. Furthermore, clarifying its role in injury-induced plasticity in the adult is of primary importance for regenerative studies. We showed here that silencing GAP-43 in the IO profoundly affects the morphology of CFs in adult rodents, leading to an atrophy of their tendrils, a retraction of the fibre and a decreased density of varicosities. GAP-43 is associated with the activation of specific cell adhesion pathways, as in the case of neuronal L1/NCAM, which are proteins involved in axonal outgrowth and synaptic plasticity [18], [44]–[48] and are highly expressed in the IO [49], [50]. GAP-43 also interacts with cytoskeletal and presynaptic scaffold proteins. It interacts, among others, with actin to regulate its polymerization, with the microtubule associated protein MAP-2 and with brain spectrin [51], [52]. Spectrin forms a filamentous network with actin under the plasma membrane, providing support for cell morphology [53]. This is confirmed in the neuromuscular junction of *Drosophila* larva, where spectrin is required in the presynaptic compartment for maintaining synapse stability [54], [55]. It is also interesting to note that a disruption of the normal distribution of the membrane proteins PlexinB1 and Nogo receptors has been recently reported in PCs following axotomy and overexpression of GAP-43 [56]. Therefore, the atrophy induced by silencing GAP-43 suggests that GAP-43 may cooperate with cytoskeletal proteins at the membrane in maintaining axonal and pre-synaptic structures by linking them to neuronal adhesion molecules.

Several lines of evidence show that ectopic overexpression of GAP-43 induces axonal sprouting. It was shown that cerebellar PCs, which constitutively lack this protein, gain the ability to sprout in transgenic mice specifically overexpressing GAP-43 in PCs [16], [18]. Likewise, adult motoneurons exhibit a spontaneous axonal sprouting and an enhanced sprouting following axotomy when GAP-43 overexpression is induced in transgenic mice [15]. In the present study we have shown *in vivo* that downregulation of GAP-43 expression is sufficient to largely prevent CF collateral sprouting induced by subtotal lesion of the IO. This demonstrates that, in this model of injury-associated axonal growth, a single molecule can affect the regenerative programme in the adult brain. However, we have also observed a few CFs in which the regenerative potential was preserved, although the overall extension of sprouting was significantly reduced. This raises the possibility that in these fibres a residual expression of GAP-43 may be sufficient to support sprouting. Alternatively, other pathways may be active in these neurons and compensate GAP-43 loss [6] such as the GAP-43 related protein MARCKS (Myristoylated Alanine-Rich C-Kinase Substrate), expressed at high levels in IO and similarly involved in promoting axonal growth [8]. A further possible interpretation relies on the heterogeneity of olivary neurons in overexpressing plasticity-related proteins in reaction to axotomy [57], raising the possibility that GAP-43 may not be necessary for sprouting in all neurons of the IO.

The experiments presented here demonstrate that in the adult brain GAP-43 plays a role in the maintenance of CF axonal structure under physiological conditions. We also induced partial degeneration of the IO by 3-AP injection, an approach used for studying structural plasticity in the cerebellum. It is remarkable to observe that specific downregulation of GAP-43 almost completely abolished collateral sprouting of the CF. This provides new data about the role played by GAP-43 in regeneration of the nervous system following injury. Axonal degeneration and synaptic loss also have a relevant role in several neurodegenerative diseases, including amyotrophic lateral sclerosis, spinal muscular atrophy, glaucoma, Alzheimer's disease, Parkinson's disease and multiple sclerosis [58]–[60]. Finding effective pharmacological treatments for modulating the expression or the activity of GAP-43 may contribute to sustain axonal structures, preventing axonal dying back in neurodegenerative diseases.

## Materials and Methods

### 4. Design and cloning of shRNA

Five candidate shRNAs were designed to specifically silence rat *Gap-43* gene (NM_017195.1), according to the rationale established by Reynolds and colleagues [61]. Each sequence was formed by a 19 nt sense core and its antisense, separated by a loop sequence (TTCAAGAGA) and followed by a stop sequence (TTTTTGGAA) [62], [63], [64]. The following sequences were so designed: 1) GACAGAAAGTGCTGCTAAA, 2) AGCTCAAAGACGAGAAGAA, 3) GAAAGAAGCTGTAGATGAA, 4) GAACATGCCTGAACTTTAA, 5) GTCCAACAGTGTGGCTTAA.

Forward and reverse oligonucleotides were annealed and cloned into pLVTHM (kindly provided by D. Trono, Lausanne, Switzerland) downstream to the RNApolIII promoter H1. All five sequences were tested and shRNA4, targeting both rat and mouse *Gap-43*, was chosen as it showed the highest efficiency. This was then sub-cloned in p207.pRRLsinPPTs.hCMV.GFP.WPRE (p207 for short, kindly provided by L. Naldini, Milan, Italy). In order to make it suitable for shRNA expression, p207 was modified (p207-SH) with the insertion of a divergent H1 promoter upstream to the CMV promoter.

As a control for short-hairpin non-specific effects, shRNA4 sequence was randomized and the absence of any possible target was checked by bioinformatics. The following SCR 19 nt sequence was obtained and cloned in the p207 transfer vector as described above: GACGAACGTATCCATATAT.

### 5. Lentiviral particles production

Lentiviral particles were produced according to previously described methods [65]. Briefly, the VSV-G-pseudotyped lentiviral particles were generated by calcium phosphate transfection of HEK293T cells with a mixture of helper and transfer plasmids [65], [66]. Following 14–16 hrs of incubation with the transfection mix, cells were washed with Dulbecco phosphate buffered solution (DPBS), and then they were grown in complete DMEM supplemented with pyruvate. Surnatant containing viral particles was harvested 40–42 hrs after transfection, filtered through a 0.45 µm Durapore Stericup unit, and concentrated by 2 ultracentrifugation steps (or only 1 for the first selection experiments). Finally, the viral pellet was suspended in DPBS with 1% bovine serum albumin (BSA) in a final volume equivalent to 1∶2,000–5,000 of the initial (1∶400 for the first selection experiments). Viral transduction efficiency was estimated on HEK239T cells measuring the rate of transduced cells by cytofluorimetry, obtaining a viral titre of 0.8–0.9*10^9^ units/µl. Viral particles were stored at −80°C in single use 2 µl aliquots.

### 6. Protein extraction and Western-blot

Rat pheochromocytoma PC12 cells (generously provided by M.E. Schwab, Zurich, Switzerland) [35], [36] were plated on 35 mm dishes coated with poly-lysine at a density of 300,000/dish. Starting from the following day they were differentiated by nerve growth factor (NGF, 100 ng/ml) for 3 days in serum free medium with 1% BSA. Every 2–3 days half of the medium was replaced with fresh medium containing NGF. Virus was added together with NGF, at a 1∶2000 dilution. Protein extracts were obtained by incubating detached cells on melting ice for 25 min in standard lysis buffer (10 mM Tris pH 7.5, 150 mM NaCl, 1% Triton-X, 10 mM NaF, 1 mM Na_3_VO_4_) added with 1% protease inhibitors cocktail (Sigma). Crude lysate was centrifuged at 15,000× g for 5 min at 4°C and supernatant was collected.

Cell extracts (20 µg) were denaturated at 96°C for 5 min and loaded on SDS-polyacrylamide gel (12%). Gels were blotted on PVDF membranes. Immunodetection was performed by rabbit polyclonal anti-GAP-43 1∶1000 (NB300-143, Novus Biological, USA), mouse monoclonal anti-vinculin 1∶1000 (Sigma) or mouse monoclonal anti-actin 1∶5000 (Sigma) as loading control and horseradish peroxidise conjugated secondary antibodies (Chemicon) and ECL-Plus (Amersham).

### 7. RNA extraction and real-time PCR

PC12 cells were plated at a density of 60,000/dish and treated with NGF and virus as described above. RNA extraction and two-step retro-transcription were performed by FastLane Cell cDNA kit (Qiagen) according to manufacturer instructions. An equivalent of 10 ng RNA was used for retrotranscription reaction. As a reference gene we chose the nuclear encoded mitochondrial ATP synthase beta polypeptide (ATP5b; NM_134364.1). *Gap-43* mRNA relative expression was measured by real-time PCR (Applied Biosystem 7900HT) using Fast-start Universal Master mix (Roche) and Universal Probe Library (Roche). Reaction was run in four replicates. Primers were designed by a dedicated online software (Roche) and checked for their amplification efficiency. Their sequences and associated fluorescent probes were the following (5′-3′): *Gap-43* Fw ACGGAGACTGCAGAAAGCA, Rev CGGGCACTTTCCTTAGGTTT, probe #63; ATP5b Fw CATGGGTACAATGCAGGAAA, Rev GGTCATCAGCTGGCACATAG, probe #77. Quantification was made by the ΔΔCt method [67], [68] on raw data exported on Excel (Microsoft).

### 8. Stereotaxic injections and partial lesion of the inferior olivary nucleus

Animals were housed according to the European Community Council Directive (86/609/CEE). The experimental protocols were designed in accordance with Italian law D.L. 116/92 and approved by the Italian Minister of Health. All efforts were made to minimize animal suffering and to reduce the number of animals used.

The method used to inject the virus into the IO was modified from Nishiyama and Linden [26]. Briefly, juvenile P19-P22 45-55g wild-type Wistar rats (Harlan) were deeply anaesthetized by an intraperitoneal injection of ketamine/xylazine and placed in a stereotaxic device. The dorsal neck muscles were retracted to expose the dura over the foramen magnum, and an opening was made to expose the brainstem. A borosilicate capillary (Sutter Instruments) was connected to a picospritzer (Parker Inst, USA) and front filled with 1.5 µl of lentiviral suspension. Injections were made unilaterally starting from the midline, at a depth of 2.2–2.3 mm. The capillary was set at an angle of 46° from vertical on the sagittal plane and 5° from the midline on the horizontal plane. A volume of viral suspension of ∼0.5 µl was delivered over a 6–12 min period, while the capillary was left in place for another 15 min before it was withdrawn.

For the different proportions of the head bones in older rats, exposure of the dorsal side of brain stem was technically not possible in a normal stereotaxical apparatus in these animals. However, this method of injection was easily adapted to adult mice (FVB, nine-twelve weeks old, Harlan) with few minor adjustments (coordinates for the injections: 55° from vertical, 7° from the midline, 1.6 mm depth; injected volume: ∼0.3 µl).

To induce a subtotal lesion of the IO rats were treated with an intraperitoneal injection of 3-AP (65–75 mg/kg) followed 3 hrs later by 15 mg/kg harmaline and 300 mg/kg nicotinamide 1.5 hrs later [69].

### 9. Immunohistochemistry

Immunohistochemistry was performed as previously described [70]. Briefly, three weeks after viral injection or after 3-AP administration rats were deeply anaesthetized and intracardiacally perfused with ice-cold 4% paraformaldehyde. Brains were post-fixed for at least 4 hrs at 4°C and equilibrated with 30% sucrose overnight for cryoprotection. Thirty micrometer-thick sagittal or 50 micrometer-thick coronal sections were cut using a freezing microtome, then permeabilized in PBS with 1% Triton-X. Sections were pre-absorbed with 10% normal donkey serum solution for 1 hr at room temperature and incubated overnight at +4°C with the following primary antibodies: mouse monoclonal anti-calbindin D28K 1∶2000 (Swant, Switzerland), rabbit polyclonal anti-GAP-43 1∶1000 (Novus Biological, USA) and rabbit polyclonal anti-VGLUT2 1∶500 (Synaptic Systems GmbH, Germany). After washing, sections were incubated 2 hrs at RT with 1∶200 Cy-3-conjugated donkey anti-rabbit (Jackson ImmunoResearch, USA) and Alexa647-conjugated donkey anti-rabbit (Invitrogen).

### 10. Confocal microscopy and image analysis

Images from immunolabelled samples were acquired with a 63× oil immersion objective and a Leica confocal imaging system (Leica TCR SP5) and processed using NIH ImageJ software [71]. GAP-43 *ex-vivo* expression was analysed on brain slice images acquired with a reduced pinhole (0.75 PAU) and using the same settings, including laser power, photomultiplier gain and offset (z-step: 0.5 µm). GAP-43-specific signal in transduced CFs was isolated and identified within each optical section for its colocalization with GFP. It was then projected on the z-axis and quantified.

The morphology of isolated GFP-labelled CFs was analyzed on images acquired from at least 3 animals per group, using always the same settings with only minor adjustments of laser power and photomultiplier gain, depending on fluorescence levels (z-step: 0.75 µm). Series of optical sections were then z-projected and analysed by ImageJ. The total length of CF was measured by NeuronJ plugin on GFP channel [72]. These tracings were analysed by Sholl's method, positioning the first main CF branching point in the centre (radius step: 2 µm; radius span: 1 µm) [40], [41]. The median number of intersections was calculated for each CF in the interval of interest, corresponding to the distance at which the maximum number of branches was present. The number of varicosities, identified either morphologically or by VGLUT2 staining, was manually counted using ImageJ.

Following 3-AP experiments, the number of GFP-positive CFs with clear signs of sprouting was counted on a epifluorescence microscope (20× objective) on 50 µm thick cerebellar coronal sections (at least 6 per animal). The isolated GFP-positive sprouting area of each CF was acquired from coronal sections as described and measured.

Data were imported in Excel (Microsoft). Mean and standard error was calculated for all parameters of each experimental group. Differences among three groups were evaluated by ANOVA and post-hoc Tukey's HSD test. Comparisons in two-groups experiments were evaluated by Student's *t*-test.

## Supporting Information

Figure S1 **Injection of viral particles, transduction and CF labelling.** (A) An example of brainstem of a rat injected with control (GFP-only) viral suspension, as observed by combined DIC and fluorescent microscopy for GFP signal (coronal section). The injection successfully reached the IO in the brain stem and induced the expression of GFP in part of the IO (Py: pyramidal tract). (B) Representative field in the cerebellar cortex of the same animal with a GFP-labelled CF with their typical arborization (ML: molecular layer; PC: Purkinje cell layer; parasagittal section).
(TIF)

Figure S2 **Sprouting of CFs transduced with lentiviral vectors following subtotal lesion of the IO.** Projections of series of optical sections obtained by confocal microscopy from sagittal (A–B) and coronal sections (C–E) of animals injected with control viral particles (expressing only GFP) and treated with 3-AP to induce the death of most neurons in the IO (VGLUT2, red; calbindin, blue). (A–B) Two examples of isolated GFP-expressing CFs (green) that survived to a subtotal lesion of the IO and developed new branches innervating the surrounding PCs, completely devoid of their original CF innervation (visible by the lack of VGLUT2-positive varicosities around the GFP-positive CF). Arrowheads indicate points of sprouting, where the CF grows on the dendritic branch of an adjacent PC in both directions, towards the distal portions as well as towards the soma. A remarkable change is evident in CF organization, consisting in thinner stalks and fewer tendrils compared to CFs in normal conditions (A, insert). In some cases some reinnervating portions of CFs profusely branch forming a rich tendril net on the newly innervated dendritic branch (B, insert). (C–E) Three examples observed on coronal sections, showing the high variability of the extension of the reinnervation. A high number of short transverse branches are present in C and D (arrows in the inserts). In some cases a longer branch innervated a dendritic arbor far from the original one (E).
(TIF)
