# Supplementary figures and images for: Impaired Sprouting and Axonal Atrophy in Cerebellar Climbing Fibres following In Vivo Silencing of the Growth-Associated Protein GAP-43
Source: PLoS One. 2011 Jun 10;6(6):e20791. doi: 10.1371/journal.pone.0020791 (PMC3112224; doi:10.1371/journal.pone.0020791)

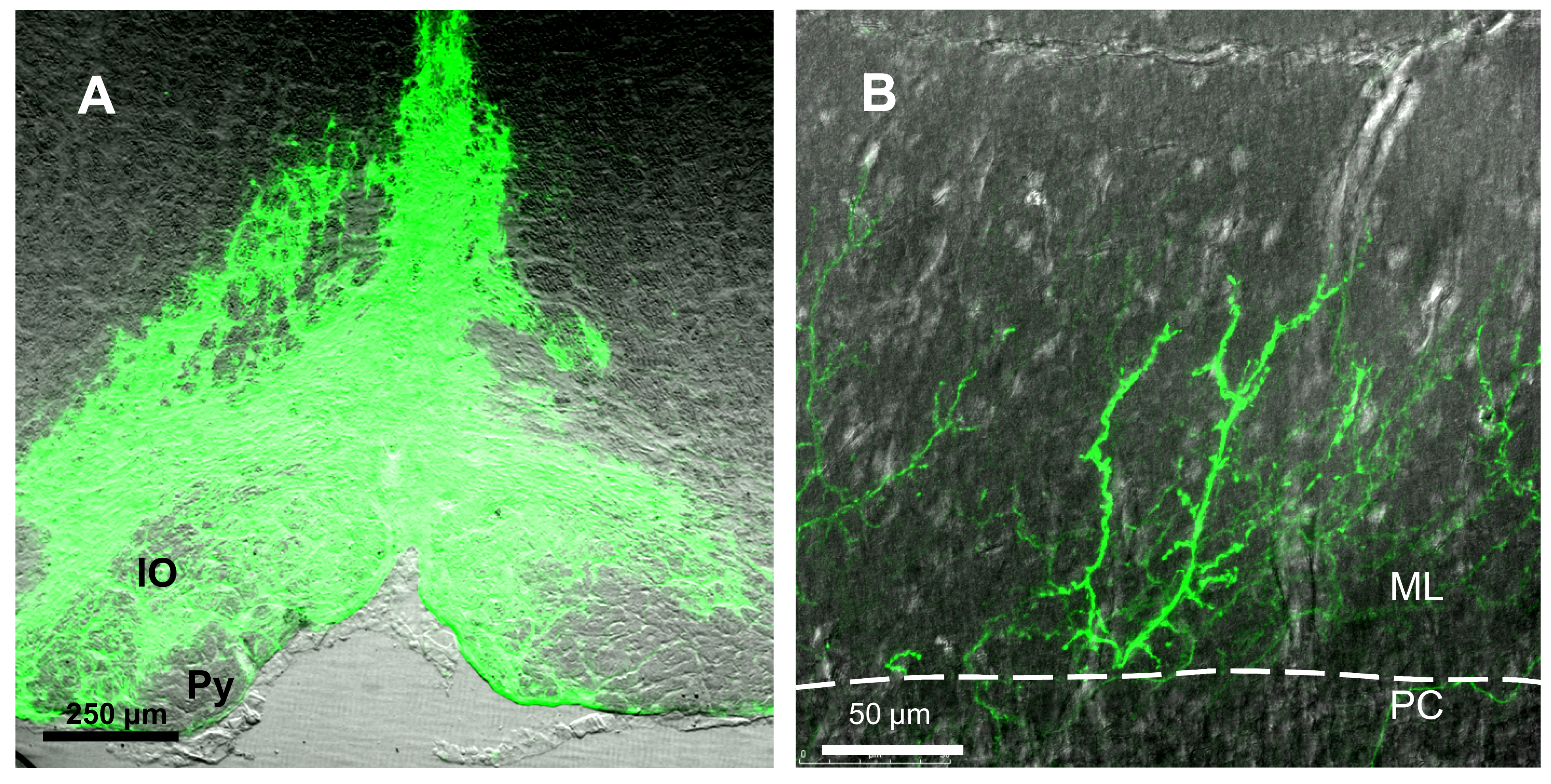

Supplement: Figure S1 — Injection of viral particles, transduction and CF labelling. (A) An example of brainstem of a rat injected with control (GFP-only) viral suspension, as observed by combined DIC and fluorescent microscopy for GFP signal (coronal section). The injection successfully reached the IO in the brain stem and induced the expression of GFP in part of the IO (Py: pyramidal tract). (B) Representative field in the cerebellar cortex of the same animal with a GFP-labelled CF with their typical arborization (ML: molecular layer; PC: Purkinje cell layer; parasagittal section). (TIF) [file pone.0020791.s001.tif]

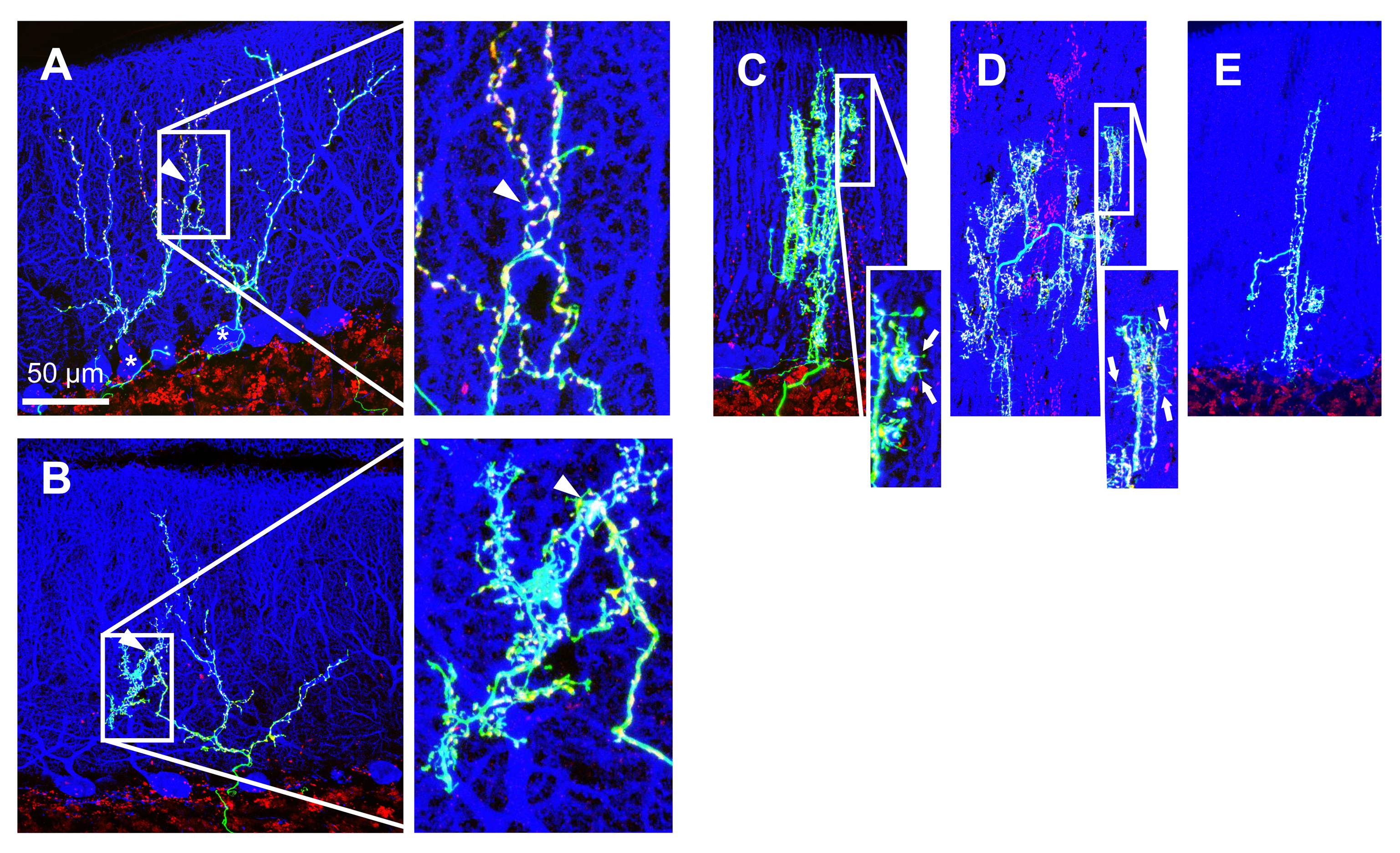

Supplement: Figure S2 — Sprouting of CFs transduced with lentiviral vectors following subtotal lesion of the IO. Projections of series of optical sections obtained by confocal microscopy from sagittal (A–B) and coronal sections (C–E) of animals injected with control viral particles (expressing only GFP) and treated with 3-AP to induce the death of most neurons in the IO (VGLUT2, red; calbindin, blue). (A–B) Two examples of isolated GFP-expressing CFs (green) that survived to a subtotal lesion of the IO and developed new branches innervating the surrounding PCs, completely devoid of their original CF innervation (visible by the lack of VGLUT2-positive varicosities around the GFP-positive CF). Arrowheads indicate points of sprouting, where the CF grows on the dendritic branch of an adjacent PC in both directions, towards the distal portions as well as towards the soma. A remarkable change is evident in CF organization, consisting in thinner stalks and fewer tendrils compared to CFs in normal conditions (A, insert). In some cases some reinnervating portions of CFs profusely branch forming a rich tendril net on the newly innervated dendritic branch (B, insert). (C–E) Three examples observed on coronal sections, showing the high variability of the extension of the reinnervation. A high number of short transverse branches are present in C and D (arrows in the inserts). In some cases a longer branch innervated a dendritic arbor far from the original one (E). (TIF) [file pone.0020791.s002.tif]
